# Microbial Intervention: An Approach to Combat the Postharvest Pathogens of Fruits

**DOI:** 10.3390/plants11243452

**Published:** 2022-12-09

**Authors:** Sargam Verma, Lucas Carvalho Basilio Azevedo, Jyoti Pandey, Saksham Khusharia, Madhuree Kumari, Dharmendra Kumar, Kaushalendra, Nikunj Bhardwaj, Pratibha Teotia, Ajay Kumar

**Affiliations:** 1 Department of Biotechnology, Noida International University, Noida 203201, India; 2 Instituto de Ciências Agrárias, Universidade Federal de Uberlândia, Campus Glória—Bloco CCG, Santa Mônica 38408-100, Brazil; 3 Department of Biochemistry, Singhania University, Jhunjhunu 333515, India; 4 Kuwar SatyaVira College of Engineering and Management, Bijnor 246701, India; 5 Indian Institute of Science, Bengaluru 560012, India; 6 Department of Zoology, C.M.B.College, Deorh, Ghoghardiha 847402, India; 7 Department of Zoology, Pachhunga University College Campus, Mizoram University (A Central University), Aizawl 796001, India; 8 Department of Zoology, Maharaj Singh College, Maa Shakumbhari University, Saharanpur 247001, India; 9 Department of Postharvest Science, Agricultural Research Organization (ARO)—Volcani Center, Rishon Lezion 7505101, Israel

**Keywords:** fruit microbiome, microbial intervention, biofilm, quorum sensing, postharvest management of fruits

## Abstract

Plants host diverse microbial communities, which undergo a complex interaction with each other. Plant-associated microbial communities provide various benefits to the host directly or indirectly, viz. nutrient acquisition, protection from pathogen invaders, mitigation from different biotic and abiotic stress. Presently, plant-associated microbial strains are frequently utilized as biofertilizers, biostimulants and biocontrol agents in greenhouse and field conditions and have shown satisfactory results. Nowadays, the plant/fruit microbiome has been employed to control postharvest pathogens and postharvest decay, and to maintain the quality or shelf life of fruits. In this context, the intervention of the natural fruit microbiome or the creation of synthetic microbial communities to modulate the functional attributes of the natural microbiome is an emerging aspect. In this regard, we discuss the community behavior of microbes in natural conditions and how the microbiome intervention plays a crucial role in the postharvest management of fruits.

## 1. Introduction

Fresh produce, including fruits, consists of essential dietary components of daily human life, which can be consumed either as raw materials or after processing. This fresh produce is the prime source of vitamins, minerals, fiber, iron, antioxidants, which also serve as essential growth factors and stimuli for the various enzymatic reactions related to normal human growth and physiology [1]. In the current conditions of the pandemic, to enhance the immune response, the consumption of fruits and vegetables has been highly recommended. According to the World Health Organization (W.H.O.), an average 400–500 g of fruits or vegetables/day has been recommended to prevent major human diseases such as hypertension, stroke, and cardiovascular disease [2,3]. This dietary behavior is close to what the human species has done for most of its existence.

The development of fruit is a coordinated transformation process from the ovary of flowers to fruits, which is completed through crossing different growth stages such as fruitlets, maturation and ripening. The development of fruits encompasses variation in the sugar, minerals, acids, etc., by changing different biochemical or metabolic processes [4]. These metabolic and physiological changes result in softening, fragrance development, color and fruit firmness [5]. However, after harvesting, the fruits are still alive until a certain period, and numerous hard-to-control biochemical processes continue, which result in quality deterioration during the postharvest handling and transformation [6].

Postharvest storage is a common practice used for the long-term storage of fruits or fresh produce to prevent deterioration by controlling pathogens and delaying the ripening process. However, fruits are highly susceptible to pathogenic attacks after harvest due to a considerable amount of nutrients, water content and low pH [7]. Currently, approximately 50% of the total fresh produce in developing countries and 25% in developed countries have been facing the risk of postharvest losses either due to improper handling, transportation, storage or postharvest pathogen attacks [1,7]. In most fruits, fungal pathogens are the prime factor responsible for the development of rots, which can emerge during transportation, handling and storage conditions.

The emergence of a pathogen causes a substantial loss of fruits and also alters the texture or quality of those fruits. Additionally, the consumer’s health may be negatively impacted by their consumption in multiple ways by their secretory mycotoxin [7,8]. Chemical fungicides, however, have been widely used around the world to control postharvest infections and disease and to maintain the firmness and freshness of fruits. [1].

However, high pesticide residue levels in fruits and the emergence of chemical-resistant pathogens are a global concern for food security that severely affects consumer health and the environment. In this context, the utilization of a microbial antagonist appears to be a suitable alternative to chemical fungicides and has seen its practice increase throughout the world [8,9]. Currently, some of the biopesticides made up of different bacteria and yeast strains, such as *Avogreen*, *Amylo-X*, *Biosave*, *Boniprotect*, *Candifruit*, and *Noli*, are marketed in different countries for the postharvest control of pathogens [9]. One aspect of this is the advantageous change in the fruit microbiome that may result from the beneficial microbial strain application.

In this review, we summarize the aspect of the postharvest disease management of fruits by modulating the natural fruit microbiome. The action mechanism of a microbial antagonistic in postharvest disease management is disclosed. In addition, the cooperative and competitive behavior among microbial communities and their possible application in postharvest pathogen management are briefly discussed.

## 2. The Fruit Microbiome: Composition and Community Structure

Plants harbor diverse microbial communities, which play a crucial role in the health and physiology of the host plant [10]. Since the long-term and intimate association between the plant and inhabitant microorganism are so strong and related throughout their life cycle, this association is referred to as the second genome and considered as a single entity, termed as a holobiont. The microbial–plant interactions act coherently, either in positive or negative ways, during the regulation of plant growth and development, and also provide stability over the long course of evolution under biotic and abiotic stress conditions [11].

Nevertheless, being immobile entities, plants evolve their mechanisms to cope with different stresses, but they also rely on their associated microbiota for survival and protection [10]. Thus, the functioning of the plant ecosystem is influenced by the associated microbiome, and could be expressed as a balance of their positive and negative correlation. Plants and the microbiome share a complex relation, and their exact molecular functioning is unclear. However, various direct and indirect mechanisms such as nutrient acquisition, phytohormone modulation, antioxidative enzyme production, disease suppression, the induction of systemic resistance, and volatile production are well-studied mechanisms that are likely involved with the growth promotion and phytopathogen management of plants [1,12]. On the other hand, the plant system provides shelter and nutrients/metabolites to the microbial counterpart for their growth and protection from invaders.

Still, we are only able to culture a very low fraction of microbial communities in laboratory conditions [13]. However, the most advanced technologies such as next-generation sequencing (NGS), metabarcoding, and metabolomics help in exploring microbial communities. The latest metagenomics studies explored the hidden potential of microbiome functioning and opened a way to their sustainable use for yield enhancement and plant protection. Various published reports showed the efficacy of microbiome functioning and employed them as biofertilizers, biocontrol, or biostimulants [12].

The microbial communities present on the plant surface or inside the plant tissue are of a diverse nature but dominated by three major bacterial phyla: *Proteobacteria, Actinobacteria* and *Bacteroidetes*, and *Ascomycota* and *Basidiomycota* are the predominantly found fungal groups [14]. In previously published reports, authors reported the microbial composition of different fruits such as the strawberry as being dominated by the *Actinobacteria, Alphaproteobacteria, Gammaproteobacteria* bacterial groups, and the *Sordariomycetes*, *Dothideomycetes*, *Leotiomycetes*, *Agaricomycetes* fungal groups [15]. Further, Abdelfattah et al. [13] briefly reported the microbial composition of the apple in a global study and found *Proteobacteria, Firmicutes* and *Actinobacteria* as the dominant bacterial phyla and *Ascomycota* followed by *Basidiomycota* as the most prevalent fungal phyla. However, the microbial composition of the plant depends upon several factors, including host genotypes, plant organs, seasons, developmental stages and environmental factors [10]. In a recent study, Zhimo et al. [16] briefly reported the apple surface microbiome of three different apple cultivars during different developmental and postharvest storage time intervals and found differences in both the bacterial and fungal communities. During different developmental and storage time intervals, the abundance of microbial genera such as *Pseudomonas*, *Pantoea*, *Aureobasidium*, and *Vishniacozyma* were decreased, while *Cladosporium, Stemphylium*, and *Alternaria* were increased in all three cultivars. However, some differences were also found in the microbial composition among all three cultivars. A study by Kecskeméti et al. [17] reported epiphytic microbial communities of ripening grape clusters and found a near to similar composition on the carposphere grown on conventional, organic, and biodynamic plots. However, a significant variation in the abundance of two species, *A. pullulans* or *A. alternate*, was observed in two different years’ samples.

### Microbiome Change in Response to Environmental Factors

The plants host a unique microbial composition that is a subset of the microbiota present in the ambient environment. As a result, a multistep model of the plant microbiome assembly was developed, in which certain environmental microorganisms were drawn to plant surfaces as epiphytes, and later some of them populated to the interior of plant tissue as endophytes [18]. This significant impact of plant habitats on microbial populations showed that microbial communities present on the plant surface adapted to the particular conditions provided by the plant hosts. The selection of specific microbial taxa by plants and their environment significantly contributes to the formation of the associated plant microbiome. Plants face environmental fluctuations that result in variations in plant secretions, which lead to variations in their structure and microbial compositions [19]. The plant-associated microbes also vary with diiferent environmental factors such soils, solar radiation, climate, location, rains, and farming practices [18]. The soils where plants develop are the primary source of root microbe recruitment. Numerous studies have reported that soil type is a key component for the construction of the root microbiome. However, different soil parameters such as types of soil, pH, the C/N ratio, etc. also play a determinant role in shaping the root microbial community [18,19]. In a brief study, Compant et al. [20] elucidated that changing climatic conditions led to variation in the root exudation and physiology of plants. This affects the amount and composition of root exudates and the availability of nutrients and signal compounds; all these factors affect microbial compositions. Furthermore, Ho et al. [21] reported that elevation and lower temperature can also affect the microbial composition by changing the amount of root exudates. Rainfall also induces variation in the microbial composition. In a study, Allard et al. [22] reported a temporary microbial shift in the epiphytic microbial composition of cucumber and tomato fruits after rainfall. Further, in a study, Louzada Pereira et al. [23] reported that the amount of sun radiation can alter the composition of internal metabolites, which can lead to stress situations that may affect the microbial composition.

The microbial composition of fruits present above (epiphytes) or inside (endophytes) also depends upon several factors, including host genotypes, season, tissue location, exudates, and the surrounding environment. As an example of microorganism populations modulated by plants, the rhizosphere is a soil region where root exudates play an essential role in the recruitment and assembly of microbial communities [10,11]. Fruits can be considered as the second most dynamic region of the plant after the rhizosphere, where a continuous shift in the microbiome can be observed from the petiole to the flowers, as well as in fruiting, harvesting and different postharvest stages. The constant physiological and metabolic changes inside the fruits lead to the variation in sugar contents and other metabolites that affect the colonization and transmission of endophytes and the assembly pattern of the epiphytic microbiome [24]. The colonization of endophytes to the host is facilitated by horizontal, vertical or mixed transmission. Horizontal transmission is mediated by the environment, wind, or the surroundings, whereas vertical transmission refers to parental means such as pollen grains, seeds, etc. However, horizontal transmission is considered as one of the influencing factors in the epiphytic microbiome structure. As the fruits are developed from the flower part, a certain fraction of endophytic microbial communities is transmitted through vertical means and show a similarity with the composition of the other components [25]. The composition of the microbiome varies even in the different compartments of the fruits, and depends upon several internal or external factors such as genotypes, season, temperature, etc. [26].

## 3. Action Mechanism of Microbial Antagonist in Postharvest Pathogen Management

Recently, the fruit microbiome, irrespective of epiphytic or endophytic microflora, has been frequently employed for the postharvest pathogen control of fresh produce. Wisniewski and Droby [27] briefly described the functional behavior of the fruit-associated microbiome in the biocontrol management system and suggested that the inhabitant microbial strains or the consortia can be used for enhancing fruit shelf life and phytopathogen control during postharvest storage.

However, for the effective management of phytopathogens, it is necessary to understand the molecular action mechanism of biocontrol agents during invasion or the association between pathogens and biocontrol agents. The most common action mechanisms of biocontrol during postharvest pathogen control are the competition for nutrients and space, mycoparasitism, volatile production, biofilm formation, and the induction of systemic resistance. In addition to quorum sensing, oxidative bursts are also mechanisms used by biocontrol agents during phytopathogen control [28,29].

In the previously published reports, several authors provided approaches for preventing postharvest pathogens. For instance, Janisiewicz et al. [30] reported on the antagonistic fungal-like yeast strain *Aureobasidium pullulans*, which prevents the disease *Penicillium expansum* from growing in fruit juice; their potential mechanism was the competition for resources. As demonstrated during the suppression of the pathogen *Rhizopus stolonifer* by the bacterial antagonism *Enterobacter cloacae* on the peach, Janisiewicz and Korsten [31] noted that direct contact between the antagonist and pathogen is essential for the mechanism of competition for nutrients and space. Furthermore, Zhu et al. [32] reported that the biocontrol yeast agent *Yarrowia lipolytica* has a better ability to grow on the wounded surface of mandarin than pathogens *Penicillium digitatum* and *Penicillium italicum* at the temperature of 20 °C and 4 °C, which has further attributed to the significant biocontrol efficacy and decay control in mandarin fruits. Similarly, Wang et al. [33] observed the rapid colonization of the biocontrol agent *Metschnikowia citriensis* in the wound tissue of citrus and tightly attached to their surface, which led to competition for nutrients and space with the pathogen *Geotrichum citri-aurantii*.

Mycoparasitism is another common mechanism of microbial antagonism, which is driven by the breakdown of pathogenic cell walls by the synthesis of enzymes such as cellulase, glucanase, and chitinase [34,35]. Nowadays, small amounts of chemical fungicides are added along with the microbial antagonist to enhance the response of the biocontrol. For instance, the pathogen *Fusarium oxysporum* has been controlled by using the antagonist *Trichoderma asperellum* and the fungicide hymexazol together. In the investigation, *T. asperellum’s* mode of pathogen suppression was hyperparasitism in both single and combined applications, and the efficacy was determined to be maximum during the combination of *T. asperellum* with hymexazol at a lower concentration. [24]. The application of biocontrol agents, however, resulted in an increase in pathogen resistance; this process is known as induced or acquired resistance. In contrast, systemic acquired resistance (SAR) and induced systemic resistance (ISR) are two prominent systemic resistances that are the subject of extensive research in plant–microbe interactions [36,37]. Protein expression patterns and phytohormonal signals are used to distinguish both of the induced resistances. However, the invasion of a necrotizing infection activates systemic resistance, which is accompanied by the salicylic acid pathway [38]. The ISR is driven by non-pathogenic bacteria and involves the ethylene and jasmonic acid signaling pathways, which successfully regulate the plant pathogens [39,40,41].

Volatiles are small diffusible organic compounds with low molecular weights that are responsible for communication between the inter/intra-microbial species or bicontrol and even pathogens. The composition of volatiles can vary with microbial strains, host genotype, growth media and the surrounding environment [42,43]. The control of phytopathogen growth by microbial volatiles also plays a crucial role in the postharvest management of fruits. Chen et al. [44] reported that volatiles generated by *Bacillus subtilis* effectively controlled the germ tube elongation and germination of the *Botrytis cinerea* spore. Similarly, volatiles emitted by *Candida intermedia* controlled the *Botrytis* fruit rot of the strawberry. The volatiles secreted by *Bacillus amyloliquefaciens* showed antifungal activity and controlled the pathogen decay of the cherry.

## 4. Collaborative Interactions among Plant Microbiota

**Figure 1:**
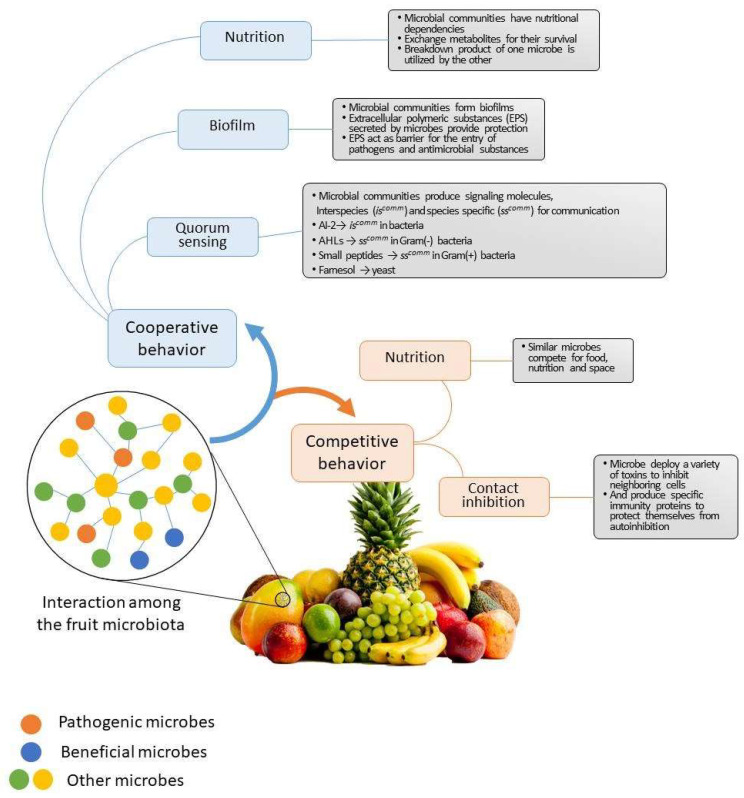
The cooperative and competitive behavior of microbial communities.

Plants are inhabited by a variety of microorganisms, and their interactions with one another are intricate. Thousands or millions of microorganisms coexist in both natural and artificial habitats as a consortium. Each microbial strain has a unique set of metabolic capacities, yet they all work together to exhibit community-level characteristics that maintain stability and offer resistance to biotic and abiotic challenges (Figure 1). However, very few studies have been carried to explore their actual behavior or cooperation between each other and how they influence the microbial assembly, plant fitness or health under normal or stressful conditions [45].

The nutrient requirement is the essential requirement for the survival of microorganisms, and in microbial communities, nutritional dependencies persist via the reciprocal exchange of metabolites, which extends their survival under limiting conditions [45,46]. In a brief review, Gralka et al. [47] reported the functional aspect of nutrient dependencies in microbial assembly. The metabolite secretion or the breakdown of products of the complex organic substrate by microbial species serve as a primary resource for another microbe. The release of residual secretory products in the surroundings can attract microbial communities and act as a nutritional buffet for the community. Thus, the rate of primary resource utilization and the structure of the microbial community play a crucial role in microbial assembly and microbiome research.

Another good example of the cooperation among microbial communities is the formation of microbial biofilm constructed by the microbial secretion of extracellular polymeric substances (EPS), which provide protection and act as a barrier to the entry of pathogens and antimicrobial compounds [48,49]. In a study, Mousa et al. [50] reported that the root-inhabiting endophytic strain *Enterobacter* sp. formed biofilm-mediated microcolonies over the finger millet. That acted as a physical or chemical barrier to the pathogen *Fusarium graminearum* and killed by trapping or providing a specific killing microhabitat. Klein and Kupper [51] reported biofilm formation by the antagonistic *Aureobasidium pullulans* against the pathogen *Geotrichum citri-aurantii*, the causal agent of sour rot in citrus. The potential for the antagonistic yeast is crucial for survival in the wounded sites of citrus and the deformation of pathogen hyphae.

The interaction between microbial species and their responses can be phenotypically determined. In a detailed study, Madsen et al. [52] observed the enhanced production of biofilm among co-cultured bacteria under natural conditions, while random co-culturing showed reduced biofilm production. However, the long-term coexistence of strains showed an adaptive response and showed enhanced biofilm formation [20].

The communication between diverse species in the community involves the synthesis and activation of small signaling molecules, or quorum sensing, which are used to detect different microbial strains. However, many organisms synchronize virulence factor secretion, biofilm formation, population behavior, and growth optimization using quorum sensing [53]. In general, interspecies and species-specific quorum sensing have both been recognized. Interspecies sensing is carried out by the furanosyl borate diester autoinducer-2 (AI-2), whereas species-specific sensing is carried out by N-acyl-homoserine lactones (AHLs), particularly in Gram-negative bacteria, while in Gram-positive bacteria it is carried out by short peptides [54,55]. However, quorum sensing is essential for interspecific or inter-kingdom communication [56]. Farnesol has been identified in recent investigations as a signaling molecule in the yeast *Candida albicans* and the opportunistic human fungal pathogen *Saccharomyces cerevisiae.* Farnesol, a signaling molecule, has been found to prevent the growth of biofilms, stop filamentation, and trigger the body’s reaction to oxidative stress [11]. Additionally, within the microbial community, some particular bacterial strains use the hyphae of filamentous fungi as a vector to spread or swim in that microsphere [57], which has been extensively reported and used in the bioremediation process, where specific bacterial strains use the hydrophobicity of fungal mycelia to reach faster and solubilize pollutants [58].

## 5. Competitive and Co-Exclusion Relationships among Plant Microbiota

Besides cooperative behavior among the microbial community, competition among different microorganisms is also observed, leading to the exclusion of certain microbial strains, providing stability to the community and maintaining host–microbiota homeostasis. This competitive behavior is generally observed more significantly among the phylogenetically similar microbes, mainly for food, nutrition resources and space [59,60]. In addition, the inhibition of specific microbial communities by contact inhibition or the secretion of antimicrobial compounds are significant factors of competition. Generally, diverse microbial communities show higher protection from pathogen invasions. In a study, Wei et al. [61] briefly described how bipartite competition for the resource is a better predictor of invasion resistance than the diverse resident microbial community in controlling *Ralstonia solanacearum* in microcosms as well as the rhizosphere of the tomato plant. In plant-associated microbial communities, numerous microbes secrete different metabolites and antimicrobial compounds that play a significant role in the growth inhibition of pathogenic or opponent microbes [62]. In a study, it was reported that these secondary metabolites remained inactive or silent in the pure culture or became active either in co-culture or community-level conditions [63]. In a study, Netzker et al. [64] reported the interaction between *Aspergillus nidulans* and *A. fumigatus* with *Streptomyces rapamycinicus* required for the activation of the secondary metabolite gene. Similarly, in another study, Tata et al. [65] reported the presence of the pathogen *Moniliophthora roreri* activating secondary metabolite production in *Trichoderma harzianum.*

## 6. Microbial Intervention in Postharvest Management

It is well established that changes in microbial diversity and composition will affect the host phenotype. The foundation of microbial intervention is the incorporation of microbial strains or microbial consortia that serve particular purposes for the host plant and help to produce a desired phenotype [66]. Nowadays, the intervention of a natural microbial community by adding microbes or microbial consortia for their beneficial and efficient application in pre- or postharvest pathogen management is an emerging aspect. The synthetic microbial community offers a modified environment and nutrient status to the natural microbiome that shields pathogen invasion [67]. Mitter et al. [68] modulated the composition of seed-associated microbial communities by inoculating the endophytic strain *Paraburkholderia phytofirmans* PsJN in the flowers of parent plants. The introduction of bacterial strains enhanced wheat’s growth traits, resulting from seed microbiome modulation via vertical transmission or from parents to offspring. Zhang et al. [69] performed the repetitive inoculation of *Trichoderma asperellum* M45a, and not only controlled the soil-borne pathogen *Fusarium oxysporum* f. sp. *Niveum*, but also enhanced the concentration of sucrose, cellulose, and antioxidative enzymes. In addition, it modulated the bacterial diversity by increasing the relative abundance of plant-growth-promoting bacteria and reducing the fungal community compared to the control untreated soil. A list of epiphytic and endophytic microbial strains of fruits and their application in postharvest pathogen management and other activities are described in Table 1.

In the recent past, functional characteristics of the fruit microbiome have been manipulated by intervening biocontrol agents or microbial consortia during preharvest, harvest, or postharvest storage conditions. The intervention and manipulation of either the epiphytic or endophytic microbiome confers either cooperative or competitive behavior among the natural plant-associated microbiome, which enhances the fruit firmness, quality or control of postharvest decay and postharvest pathogens. Nevertheless, microbiome intervention anticipates the interactions with fruit diseases but varies with fruit genotype and postharvest conditions such as temperature and storage duration [88].

The induction of resistance by the inoculated or applied biocontrol agents is dependent on several factors such as the density of biocontrol agents, plant cultivars, and the nature of the pathogen, etc. For example, in a study, Ardanov et al. [89] inoculated *Methylobacterium* spp. strains against different pathogens and discovered the induction of disease resistance, which was dependent on the density of *mycobacterium* inoculum, cultivar and pathogens.

However, plant cultivars can have a significant impact on the microbial composition [90]. Regardless of whether microbial antagonists are used singly or in combination, their use has a significant effect on how the microbiome of fruits is established. Cruz et al. [91] examined the effects of using the biocontrol agents *Trichoderma harzianum*, *Beauveria bassiana*, and *Bacillus amyloliquefaciens* alone and together on the strawberry microbiome. The combined use of biocontrol agents enhanced their effectiveness while limiting the spread of the postharvest disease *Botrytis cinerea*. Additionally, the combined application altered the strawberry bacterial and fungal communities, and the combined application caused a more noticeable microbiome shift than a single application did.

Recently in a study, Zhimo et al. [92] applied the biocontrol agent *Metschnikowia fructicola* prior to harvest to assess their impact on the strawberry microbiome. The extensive metagenomic analysis revealed a significant shift in the bacterial and fungal communities associated with strawberry during preharvest, harvest or postharvest conditions. The application of *M. fructicola* significantly enhanced the composition of the bacterial community, specifically the genera *Methylobacterium*, *Sphingomonas*, *Bacillus*, and *Rhizobium*. In addition, the biocontrol application potentially plays a role in postharvest disease suppression. Similarly, Biasi et al. [93] intervened in the epiphytic microbiome of apple fruits by applying the biocontrol agent *Metschnikowia fructicola* followed by postharvest storage. The application of biocontrol agents significantly affected the apples’ surface microbiome, and the applied *Metschnikowia fructicola* remained persistent throughout the storage conditions. In addition, all the storage samples showed reduced common postharvest pathogens of apples. The application of *Metschnikowia fructicola* significantly affected the surface microbiome of apples via decreasing the richness and microbiome shift in fungal microbiota compared to the control samples throughout the storage conditions.

## 7. Future Perspective

Fruit microbiomes have emerged as a viable method for managing postharvest antagonists, but they still need to overcome various challenges and avail the use of new resources. It is always important to optimize the physico-biological growth conditions and the size of the microbial inoculum. To extend the inoculum’s shelf life, additional study is needed on a variety of encapsulation techniques, such as powder creation, liquid formulations, and recently emerging micro- and nano-encapsulated microorganisms. The research must concentrate on the isolation and screening of stress-tolerant microorganisms in order to cope with the challenging postharvest fruit circumstances. For this, a variety of microbiomes from harsh regions, microbiomes from crops or fruits that have been infected by pathogens, and microbiomes from various environmental circumstances can be screened to find bacteria that can withstand stress. Microbes that can withstand stress and that are isolated from various soil types can be effective microbial antagonists in postharvest situations.

Competition from other chemical and physical antagonistic agents and cost-cutting measures present significant challenges when applying microbial antagonists in postharvest conditions. Developing them cost-effectively with a sustained release of microbial metabolites is required for their commercial scale-up. The modification of the fruit genome, proteome, and metabolome is another significant issue that needs to be addressed before the scale-up of microbial antagonists in postharvest circumstances. Following the inoculation of microorganisms that promote plant development, several investigations have demonstrated that plant genome-wide transcriptome responses are positively modulated. Similar to this, it is important to comprehend how microbial antagonists affect the chemical reactions of fruits. Fruits must always be made safe for consumption without negatively impacting their metabolome because they are eaten fresh from the plant. The taste, nutritional value, and molecular properties of fruits must not be hampered by the microbial antagonists. The current advancements in next-generation sequencing, conventional and non-conventional metabolome engineering, and encapsulation technologies all contribute to the positive outlook for microbial antagonists in the control of fruit postharvest disease.

## 8. Conclusions

In recent years, the postharvest management of fresh produce using a microbial antagonist has been frequently employed and considered the best suitable alternative of chemical fungicides. Recently, the fruit microbiome has been observed to play a significant role in shaping the quality and quantity of fruits. Microbial antagonists employ a number of mechanisms including competition, mycoparasitism, rapid colonization and the secretion of antimicrobial volatile antimicrobial compounds for the postharvest management of fruits. Fruit microbiomes have shown a mutualistic, competitive and co-exclusion relationship with plant microbiota for their growth and survival. However, after so much advancement in NGS technologies, we can still only find less than 1–10% of microbial strains for their further applications. Thus, the primary limitation of NGS technologies is only just to explore the hidden microbiota, but their cultivation under laboratory conditions is still a challenging task. Therefore, extensive research is needed in the plant–microbiome field to isolate and screen cultivable microbiota that can be used for biocontrol agents.

## Figures and Tables

**Table 1:** Epiphytic and endophytic microbial strains of fruits and their potential application in postharvest pathogen management and other activities.

Fruits	Genera/Strains	Function	References
Epiphytic strains			
Banana	*Clonostachys byssicola*, *C. pallescens*, *Penicillium oxalicum*, and *Trichoderma harzianum*	Biocontrol agent	[70]
Banana	*Bacillus amyloliquefaciens*	Biocontrol activity against crown-rot-causing pathogens	[71]
Citrus	*Pichia anomala*, *Debaryomyces hansenii*, *Hanseniaspora guilliermondii*	Biocontrol activity against *P. digitatum*	[72]
Citrus	*Candida oleophila* and *Debaryomyces hansenii, Bacillus amyloliquefaciens*, *B. pumilus* and *B. subtilis*	Antagonistic activity against *Penicillium digitatum* and *P. italicum*	[73]
Lemon	*Clavispora lusitaniae*	Antagonistic activity against *Penicillium digitatum*	[74]
Withered grapes	*Bacillus, Brevibacillus, Curtobacterium, Micrococcus, Pseudomonas, Staphylococcus*	Antagonistic effects on grape-rotting fungi	[75]
Grape berries	*Issatchenkia orientalis*, *Metschnikowia pulcherrima*, *Kluyveromyces thermotolerans*, *Issatchenkia terricola* and *Candida incommunis*,	Killer activity against *Aspergillus carbonarius* and *A. niger*	[76]
Apple blossoms	*Pantoea agglomerans* and *Pseudomonas* spp. *Cryptococcus* spp.	Biocontrol activity against *Erwinia amylovora*	[77]
Apple	*Aureobasidium pullulans* and *Hanseniaspora uvarum*	Not mentioned	[78]
Apple	*Aureobasidium*, *Metschnikowia*, and *Rhodotorula*	Not mentioned	[79]
Endophytic strains			
Apple	*Schwanniomyces vanrijiae, Galactomyces geotrichum, Pichia kudriavzevii, Debaryomyces hansenii*, and *Rhodotorula glutini*	Biocontrol activity against *Monilinia fructigena*	[80]
Strawberry	*Sporidiobolus* sp., *Rhodotorula* sp., Pilidium concavum, *Corynespora cassiicola, Neodeightonia subglobosa, Aspergillus awamori*, and *Aspergillus* sp.	Antioxidant activity	[81]
Strawberry	*Lactobacillus plantarum*	Antagonistic activity against *Botrytis cinerea*	[82]
Strawberry	*B. subtilis*, *Enterobacter* sp., *Pseudomonas* sp.	Plant growth promotion	[83]
Guava	*Saccharomycopsis fibuligera*	Management of gray mold rot of guava	[84]
papaya	*Kocuria, Acinetobacter, Enterobacter, Bacillus Staphylococcus*	Not mentioned	[85]
Grapes	*Bacillus cereus*	Not mentioned	[86]
Jambolana	*Neofusicoccum parvum, Pestalotiopsis*	Not mentioned	[87]

## Data Availability

Not applicable.
